# GREB1L overexpression correlates with prognosis and immune cell infiltration in lung adenocarcinoma

**DOI:** 10.1038/s41598-021-92695-x

**Published:** 2021-06-24

**Authors:** Yilin Yu, Zhiping Wang, Qunhao Zheng, Jiancheng Li

**Affiliations:** Fujian Medical University Cancer Hospital, Fujian Cancer Hospital, Fuzhou, Fujian China

**Keywords:** Cancer, Immunology, Molecular biology

## Abstract

GREB1L is a protein-coding gene that is an important paralog of GREB1. However, its effects in lung adenocarcinoma (LUAD) have not been determined. Thus, we evaluated the prognostic value of GREB1L in LUAD using bioinformatics approaches. In particular, we evaluated the relationship between GREB1L and LUAD using a wide range of databases and analysis tools, including TCGA, GEO, HPA, TIMER, cBioPortal, and MethSurv. Compared with its expression in normal lung tissues, GREB1L expression was significantly increased in LUAD tissues. A univariate Cox analysis showed that high GREB1L expression levels were correlated with a poor OS in LUAD. Additionally, GREB1L expression was independently associated with OS through a multivariate Cox analysis. GSEA analysis revealed enrichment in cell cycle, immune regulation, and methylation. Moreover, high GREB1L expression was associated with poor survival. We also found that the methylation and genetic alteration level was associated with prognosis in patients with LUAD. Finally, an analysis of immune infiltration showed that GREB1L is correlated with immune cell infiltration, PD-1, and PD-L1. In summary, these results indicate that GREB1L is a potential molecular marker for poor prognosis in LUAD and provide additional insight for the development of therapies and prognostic markers.

## Introduction

Lung cancer is the leading cause of cancer death worldwide. It is reported to have the second-highest incidence of cancer in both men and women in the United States, second only to prostate cancer and breast cancer, respectively. It is also the most common cause of human cancer deaths, accounting for more than 25% of all cancer deaths^1,2^. Lung adenocarcinoma (LUAD) is the most common type of lung cancer. In recent years, the number of LUAD patients is rising due to smoking and air pollution^3^. Some studies have shown that the overall 5-year survival rate of patients with advanced LUAD is less than 15%, but if patients are treated with targeted gene-targeting therapy, their survival rate will be improved^4^. However, due to the lack of specific biomarkers of LUAD, the metastasis rate and mortality rate of LUAD patients is very high^5^. Although molecular targeted therapy has made progress in recent years, but more targets need to be identified. Nowadays, immunotherapy has been paid more and more attention from oncologists. T cell is an important medium of tumor immunity. In most tumors, T cell infiltration is a useful prognostic marker^6,7^. Therefore, the development of lung cancer treatment needs further research, especially to find potential prognostic molecular biomarkers and new targets of immune-related therapy.

GREB1L (GREB1 Like Protein) is a protein-coding gene. It is an important paralog of GREB1 (Growth Regulating Estrogen Receptor Binding 1). It plays a major role in early metanephros and genital development. Previous studies have shown that GREB1L is associated with bilateral renal hypoplasia, inner ear malformation, and deafness^8–10^. In addition, a study confirmed that the methylation level of GREB1L is related to immune response and cytolysis in gastric adenocarcinoma, suggesting that it may be a new prediction and prognostic biomarker that aids in the therapy and predicts the overall survival possibility in patients with gastric adenocarcinoma^11^. However, research is lacking on the potential function of GREB1L in LUAD. It is the first study to demonstrate the functional impact of GREB1L in lung adenocarcinoma.

In this study, we synthetically evaluated the prognostic value of GREB1L expression in patients with LUAD from the Cancer Genome Atlas (TCGA) database. Furthermore, the prognostic value of GREB1L expression in LUAD was validated using data from the Gene Expression Omnibus (GEO) databases. Additionally, we performed GSEA function and pathway analysis to gain further insights into the biological mechanism of GREB1L in LUAD pathogenesis. We also examined the correlation between GREB1L expression and genetic alteration and methylation. Finally, ssGSEA (single-sample Gene Set Enrichment Analysis) and TIMER were used to explore the relative proportions of different kinds of immune cell infiltration levels in tumor microenvironments to study the relationship of GREB1L, comprehensively analyze and discuss the possible molecular mechanism between GREB1L and tumorigenesis, and tumor-immune interactions.

## Results

### Clinical characteristics

Our data were obtained from TCGA, including 497 LUAD patients with clinical data and gene expression data. The clinical characteristics, including gender, age, number pack years smoked, T stage, N stage, M stage, pathological stage, vital status, and gene expression data were collected (Table 1).

**Table 1:** Clinical characteristics of LUAD patients in TCGA.

Clinical characteristics	Total (497)	Percentage (%)
**Gender**		
Male	228	45.9
Female	269	54.1
**Age**		
≤ 65 years old	236	47.5
> 65 years old	251	50.5
**Number pack years smoked**		
< 40	167	33.6
≥ 40	174	35.0
**T stage**		
T1	166	33.4
T2	267	53.7
T3	43	8.7
T4	18	3.6
**N stage**		
N0	321	64.6
N1	94	18.9
N2	69	13.9
N3	2	0.4
**M stage**		
M0	331	66.6
M1	24	4.8
**TNM stage**		
Stage 1	267	53.7
Stage 2	118	23.7
Stage 3	80	16.1
Stage 4	25	5.0
**Vital status**		
Dead	180	36.2
Alive	317	63.8
**GREB1L expression**		
Low	248	49.9
High	249	50.1

### GREB1L is upregulated in LUAD

**Figure 1:**
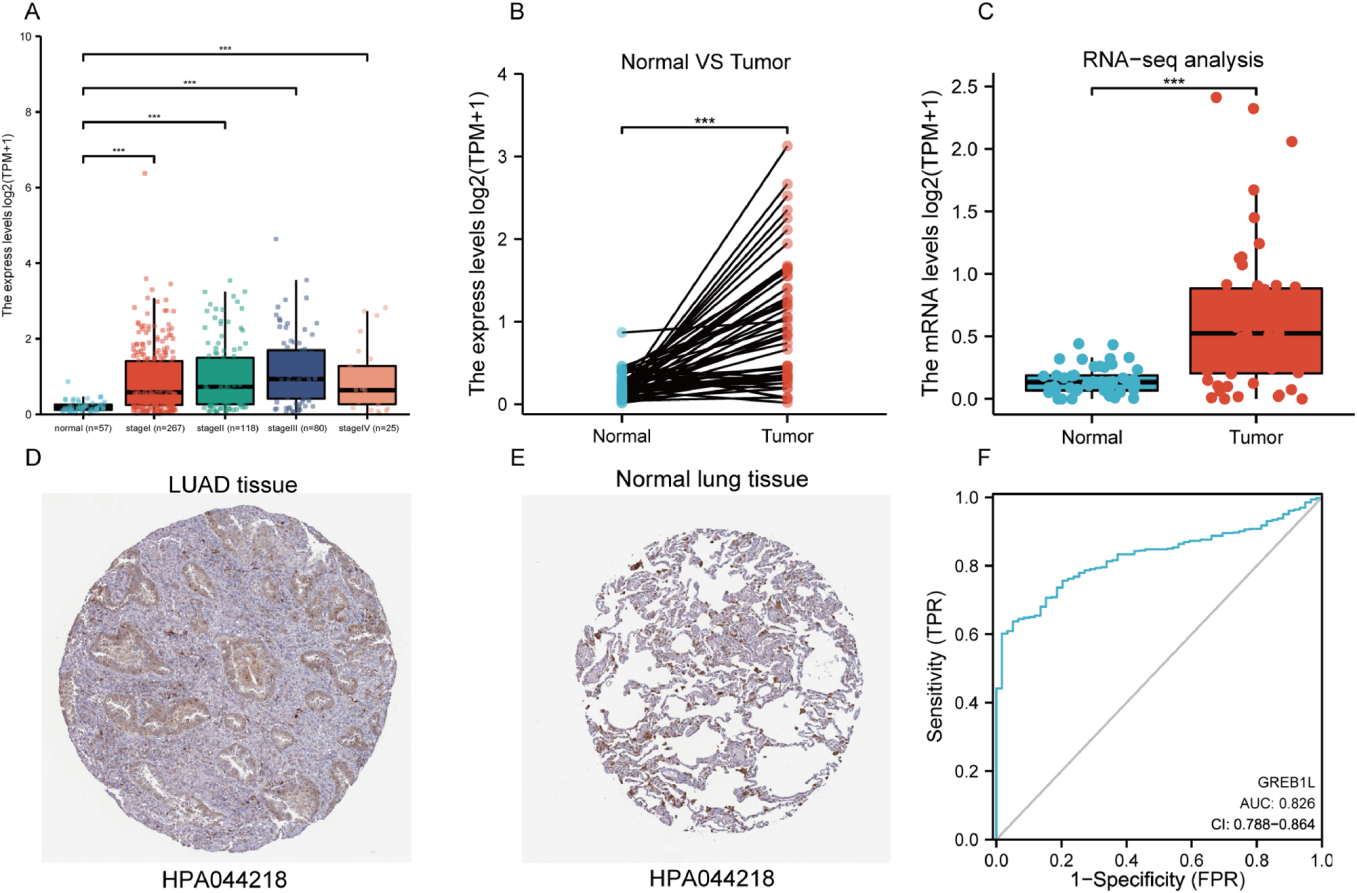
GREB1L expression levels in LUAD from TCGA data. (**A**) Expression levels of GREB1L in LUAD and normal tissue; (**B**) the expression of GREB1L in LUAD and its paired adjacent tissues; (**C**) the mRNA expression of GREB1L in LUAD was obtained from the GSE140343; (**D**,**E**) the protein expression of GREB1L in LUAD was obtained from the Human Protein Atlas; (**F**) receiver operating characteristic analysis (ROC) of GREB1L in LUAD (*p < 0.05, **p < 0.01, ***p < 0.001).

The results showed that GREB1L was overexpressed in LUAD tissues than in normal tissues (p < 0.001) (Fig. 1A). In paired samples, the expression of GREB1L in the LUAD groups was significantly higher than the adjacent normal groups (p < 0.001) (Fig. 1B). After exploring the gene expression of GREB1L in LUAD, we tried to examine the mRNA expression and protein expression patterns by the GSE140343 and the Human Protein Atlas. As shown in Fig. 1C, the mRNA expression of GREB1L was low in normal lung tissues and high in LUAD tissues. The expression of GREB1L was low in normal lung tissues, while median protein expression of GREB1L was observed in LUAD tissues (Fig. 1D,E). Besides, the ROC showed that the expression of GREB1L in LUAD was 0.826 (CI 0.788–0.864) (Fig. 1F). The differential mutated genes between patients with different GREB1L expression levels were shown in the Supplementary file 1.

### High GREB1L expression is associated with adverse outcomes in LUAD

**Figure 2:**
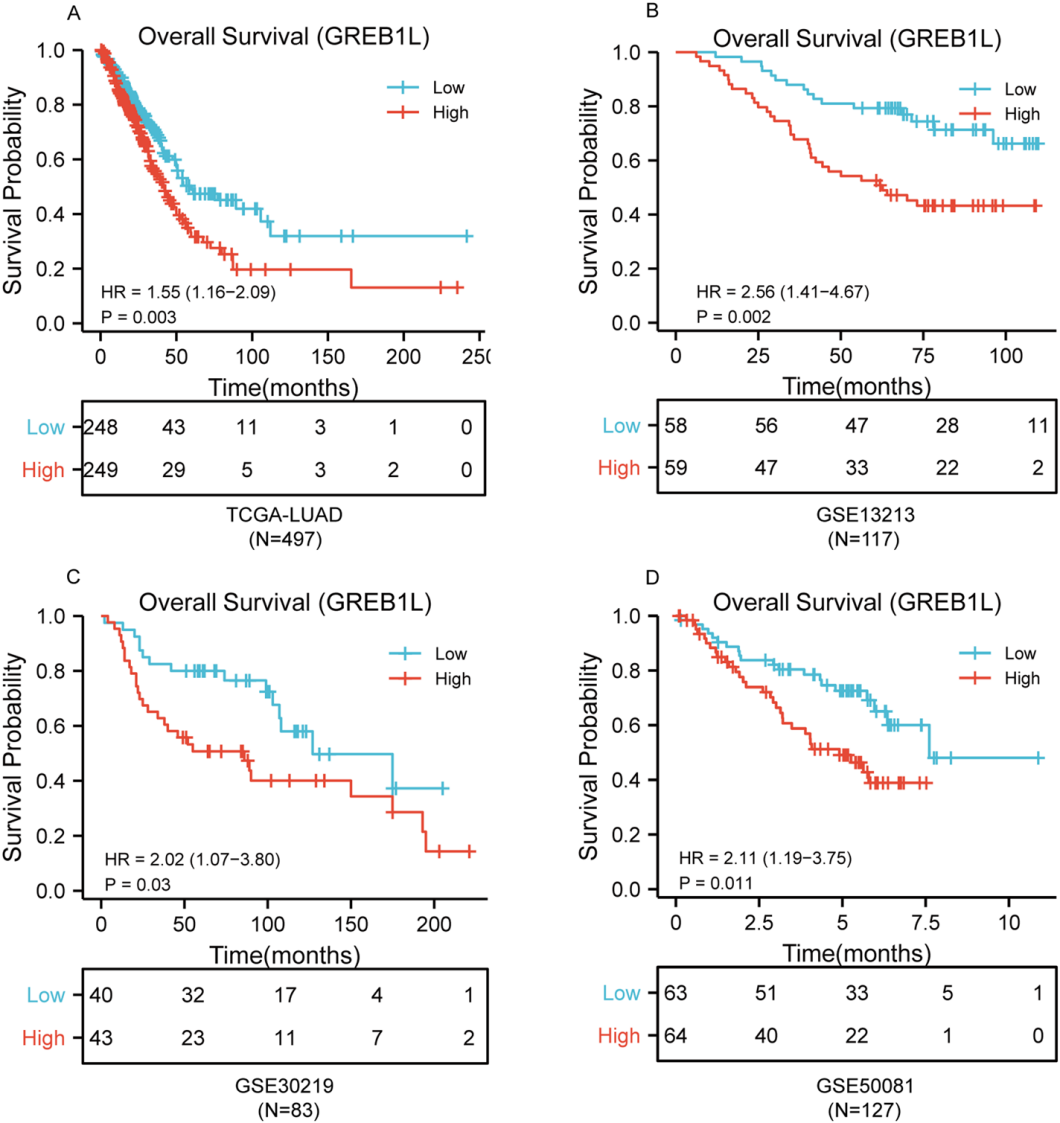
The prognostic value of GREB1L expression in LUAD. (**A**) Survival curves of OS from TCGA data (n = 497); (**B**) survival curves of OS from GSE 13213 (n = 117); (**C**) survival curves of OS from GSE30219 (n = 83); (**D**) survival curves of OS from GSE50081 data (n = 127).

In order to explore the correlation between GREB1L expression and prognosis, the expression level of GREB1L from TCGA was classified as low- and high-expression according to the median expression value. The results showed that the overall survival (OS) of high GREB1L expression was significantly poorer than low GREB1L expression (p < 0.01) in LUAD (Fig. 2A). To further validate the correlation between GREB1L expression and overall survival, we examined the GSE13213, GSE30219, and GSE50081 datasets. The results also showed that high GREB1L expression had a worse OS than low GREB1L expression (Fig. 2B–D). We next used the univariate and multivariate Cox regression model to explore the prognostic factors in LUAD. It showed that high GREB1L expression level was correlated with inferior OS. Due to the missing data of the M stage over 30% in TCGA, it was not included in the multivariate analysis. Multivariate analysis showed that GREB1L expression was an independent prognostic factor for LUAD in both TCGA and GSE13213 datasets (Table 2).

**Table 2:** The univariate and multivariate analyses of overall survival according to GREB1L expression, after adjusting for other potential predictors in TCGA and GSE13213.

Clinicopathologic variable	Total (N)	HR (95% CI)	p-value
**TCGA-LUAD**			
a.			
Gender (male vs. female)	497	0.954 (0.711–1.279)	0.752
Age (> 65 vs. ≤ 65)	487	1.221 (0.907–1.643)	0.188
Number pack years smoked (> 40 vs. ≤ 40)	341	1.026 (0.714–1.475)	0.888
T stage (T2/T3/T4 vs. T1)	494	1.678 (1.187–2.373)	0.003
N stage (N2/N3 vs. N0/N1)	486	2.274 (1.589–3.255)	< 0.001
M stage (M1 vs. M0)	355	2.129 (1.243–3.648)	0.006
Pathologic stage (stage III/IV vs. stage I/II)	490	2.629 (1.924–3.591)	< 0.001
GREB1L (high vs. low)	497	1.554 (1.156–2.088)	0.003
b.			
T stage (T2/T3/T4 vs. T1)		1.533 (1.008–2.334)	0.046
Pathologic stage (stage III/IV vs. stage I/II)		2.222 (1.576–3.132)	< 0.001
GREB1L (high vs. low)		1.453 (1.076–1.962)	0.015
**GSE13213**			
a.			
Gender (male vs. female)	117	0.736 (0.419–1.293)	0.286
Age (> 65 vs. ≤ 65)	117	1.394 (0.784–2.480)	0.258
Smoking (yes vs. no)	117	1.361 (0.774–2.391)	0.284
T stage (T2/T3/T4 vs. T1)	117	1.539 (0.866–2.735)	0.142
N stage (N2 vs. N0/N1)	117	2.810 (1.543–5.116)	0.001
Pathologic stage (stage III vs. stage I/II)	117	2.974 (1.659–5.331)	< 0.001
EGFR status (mut vs. wt)	117	1.021 (0.574–1.815)	0.945
K-ras status (mut vs. wt)	117	1.530 (0.717–3.267)	0.272
p53 status (mut vs. wt)	117	1.357 (0.764–2.412)	0.298
GREB1L (high vs. low)	117	2.564 (1.408–4.666)	0.002
b.			
N stage (N2 vs. N0/N1)		1.147 (0.261–5.037)	0.856
Pathologic stage (stage III vs. stage I/II)		2.217 (0.517–9.515)	0.284
GREB1L (high vs. low)		2.207 (1.195–4.077)	0.011

### Development of a prognostic model based on GREB1L and clinical factors

**Figure 3:**
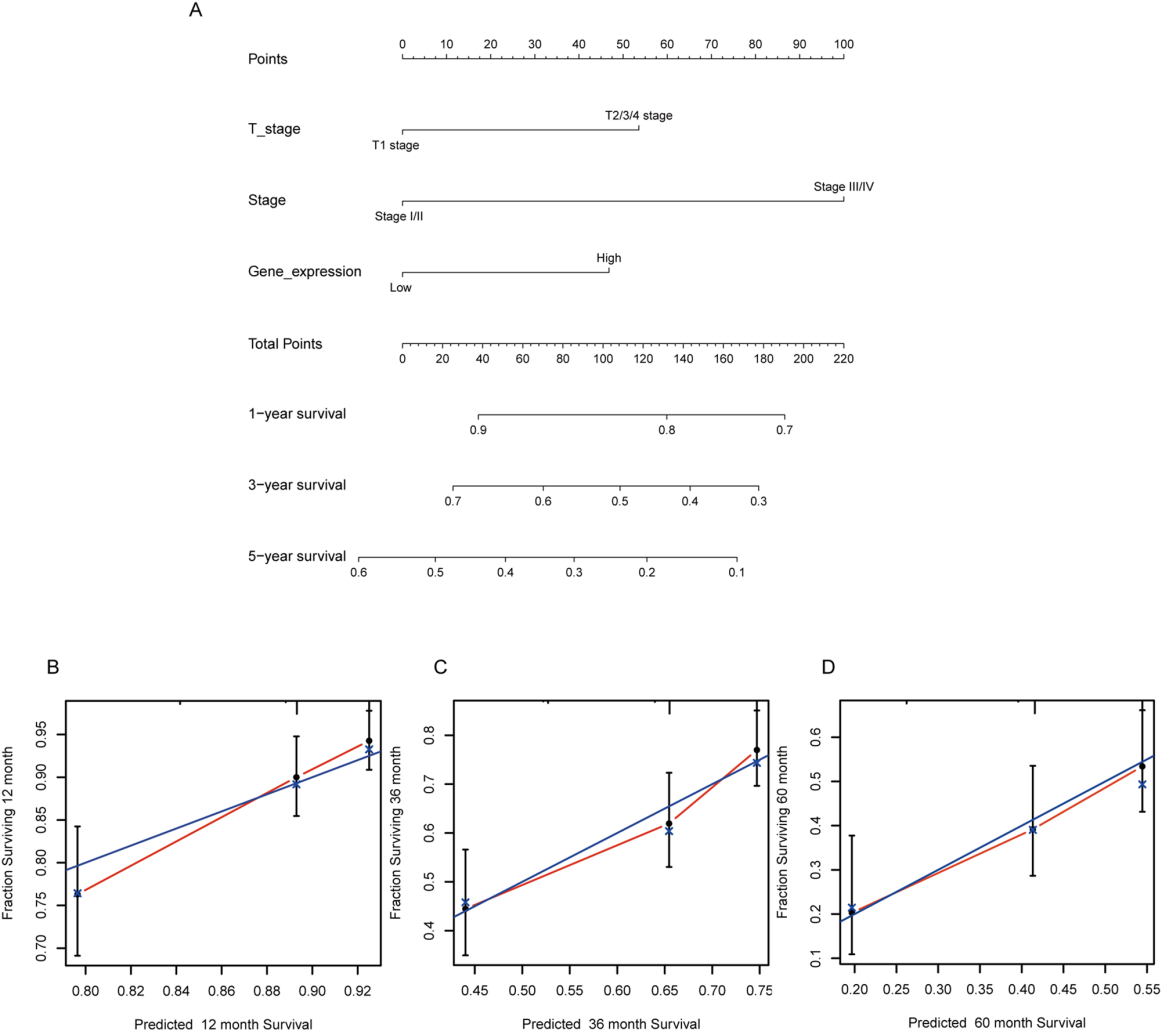
Nomogram and calibration curve for predicting the probability of 1-, 3- and 5-year OS for LUAD patients. (**A**) A nomogram integrates GREB1L and other prognostic factors in LUAD from TCGA data; (**B**–**D**) The calibration curve of the nomogram.

The multivariate analysis result indicated that GREB1L is an independent prognostic factor in LUAD. We then constructed a prediction model for overall survival by fitting the expression of GREB1L and other clinical parameters. We established a nomogram to integrate GREB1L as a LUAD biomarker (Fig. 3A). A higher point on the nomogram represented a worse prognostic factor. The calibration curve evaluated the nomogram's performance of GREB1L. The C-index was 0.6424 of GREB1L with 1000 bootstrap resamples for the nomogram (Fig. 3B–D). To sum up, this nomogram may be a model for predicting survival in LUAD with GREB1L than an individual prognostic factor.

### Function and pathway enrichment analysis by GSEA

**Figure 4:**
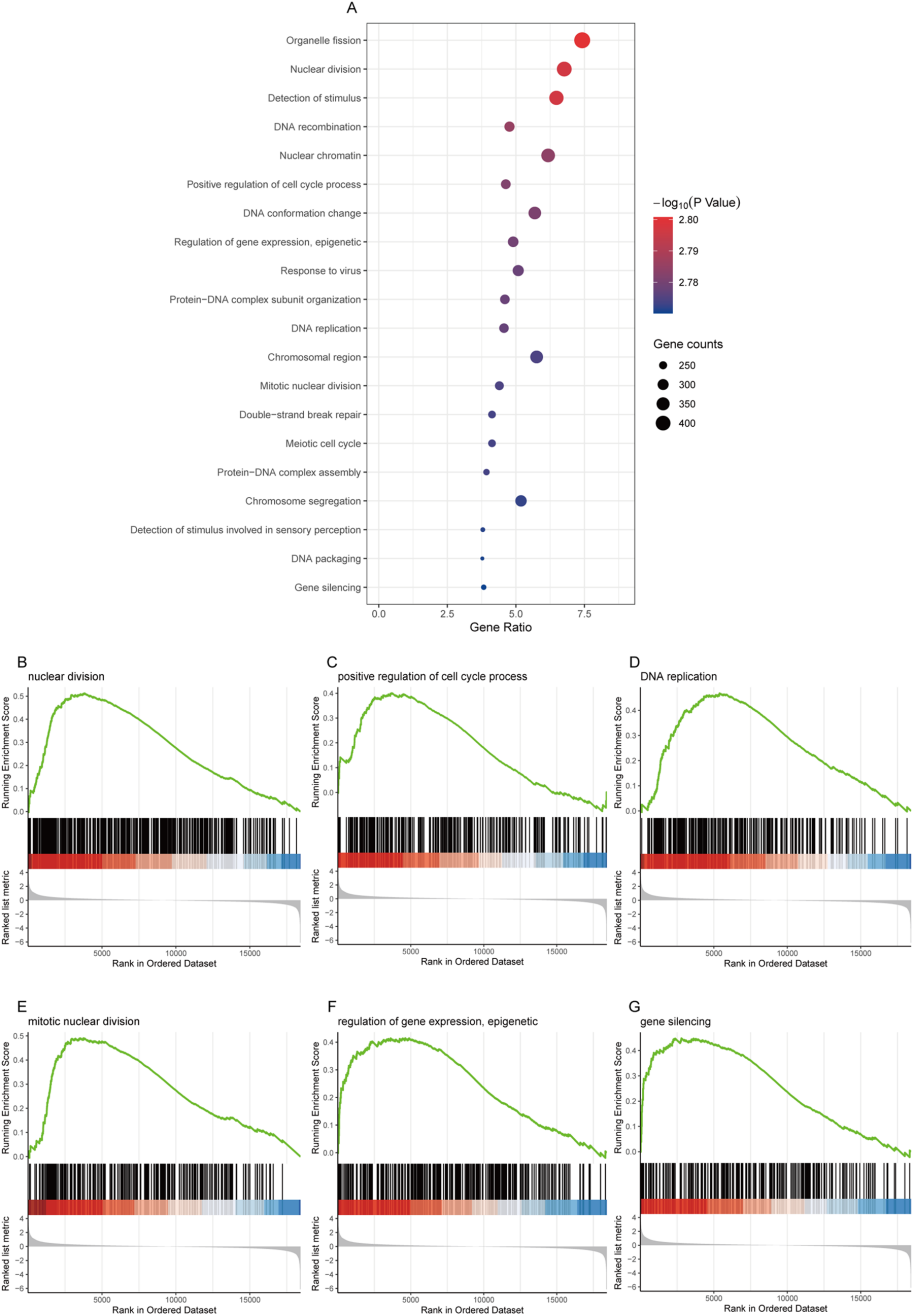
Functional enrichment of GREB1L in LUAD. (**A**) GSEA function enrichment analysis of differentially expressed genes (DEGs) in low- and high- GREB1L expression samples; (**B**–**G**) Enrichment of genes in the nuclear division, positive regulation of cell cycle process, DNA replication, mitotic nuclear division, epigenetic regulation of gene expression, and gene silencing by GSEA function analysis.

**Figure 5:**
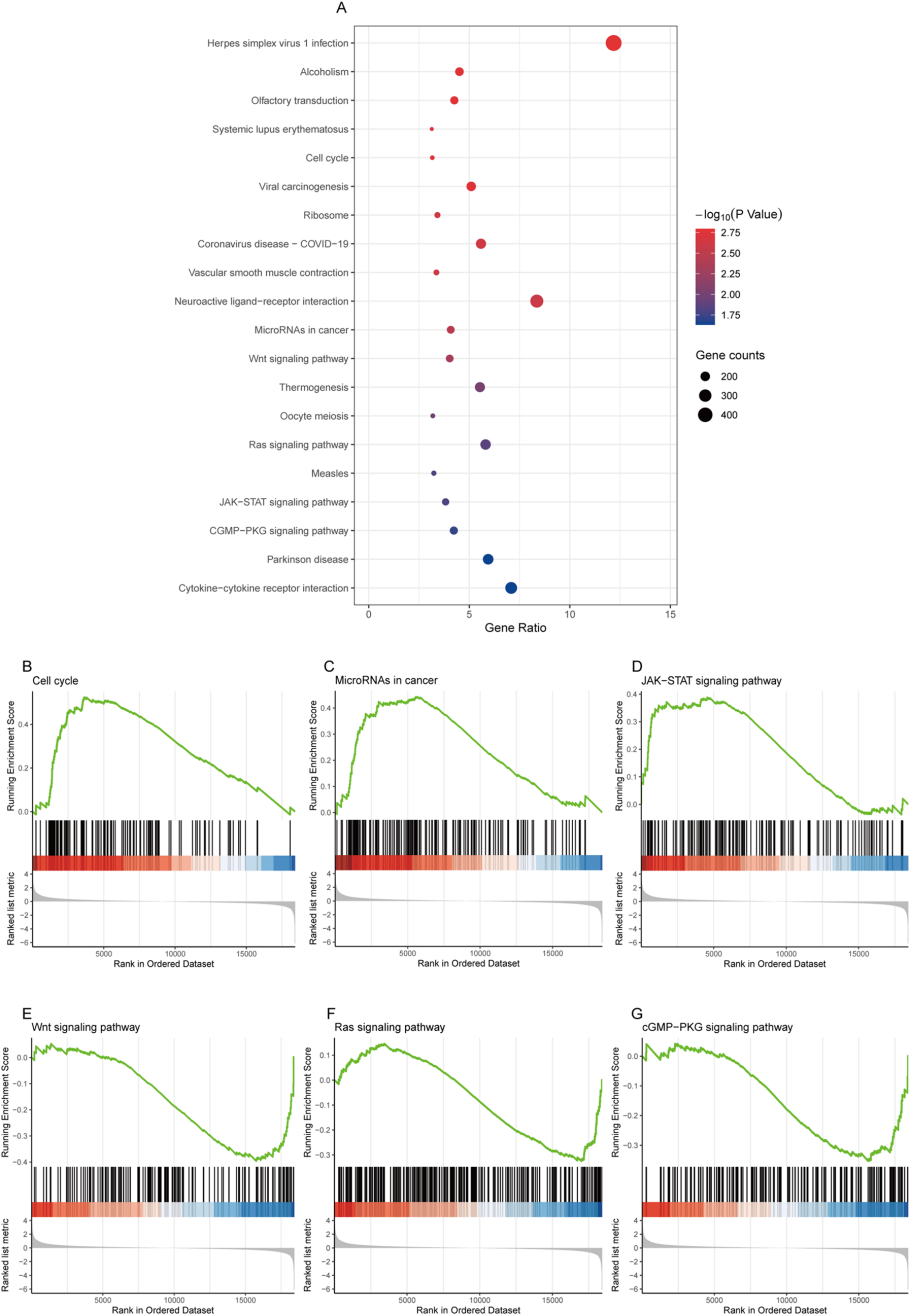
Functional enrichment of GREB1L in LUAD. (**A**) GSEA pathway enrichment analysis of differentially expressed genes (DEGs) in low- and high- GREB1L expression samples; (**B**–**G**) Enrichment of genes in the Cell cycle, MicroRNAs in cancer, JAK- STAT signaling pathway, Wnt signaling pathway, Ras signaling pathway, and cGMP- PKG signaling pathway by GSEA pathway analysis.

To elucidate the biological functions of GREB1L, we analyzed the differentially expressed genes (DEGs) between the DNA amplification and non-amplification groups. We divided patients into low- and high- expression groups based on the median GREB1L expression value. GSEA functionanalysis showed that 18 biological processes (BP) and 2 cellular components (CC) were enriched (Fig. 4A–G). The 20 GSEA function analysis were all upregulated. Among the 18 BP terms, eight were associated with cell cycle, including “organelle fission”, “nuclear division”, “DNA recombination”, “positive regulation of cell cycle process”, “DNA replication”, “mitotic nuclear division”, “meiotic cell cycle”, and “chromosome segregation”, two were associated with methylation, including “regulation of gene expression, epigenetic” and “gene silencing”. The remaining BP terms were “detection of stimulus”, “DNA conformation change”, “response to virus”, “protein-DNA complex subunit organization”, “double-strand break repair”, “protein-DNA complex assembly”, “detection of stimulus involved in sensory perception” and “DNA packaging”. The 2 CC terms were “nuclear chromatin” and “chromosomal region”. To further explore the potential biological pathways of GREB1L that promote tumor progression, we also performed a GSEA pathway analysis (Fig. 5A). The results showed that high GREB1L expression was upregulated in the Cell cycle, MicroRNAs in cancer, and JAK-STAT signaling pathway (Fig. 5B–D). However, the Wnt signaling pathway, Ras signaling pathway, and cGMP-PKG signaling pathway were down-regulated (Fig. 5E–G). Among these pathways, “JAK-STAT Signaling pathway” and “cytokine-cytokine receptor interaction” were immune-related pathways.

### Correlation between GREB1L mutation, hypomethylation, and prognosis in LUAD

**Figure 6:**
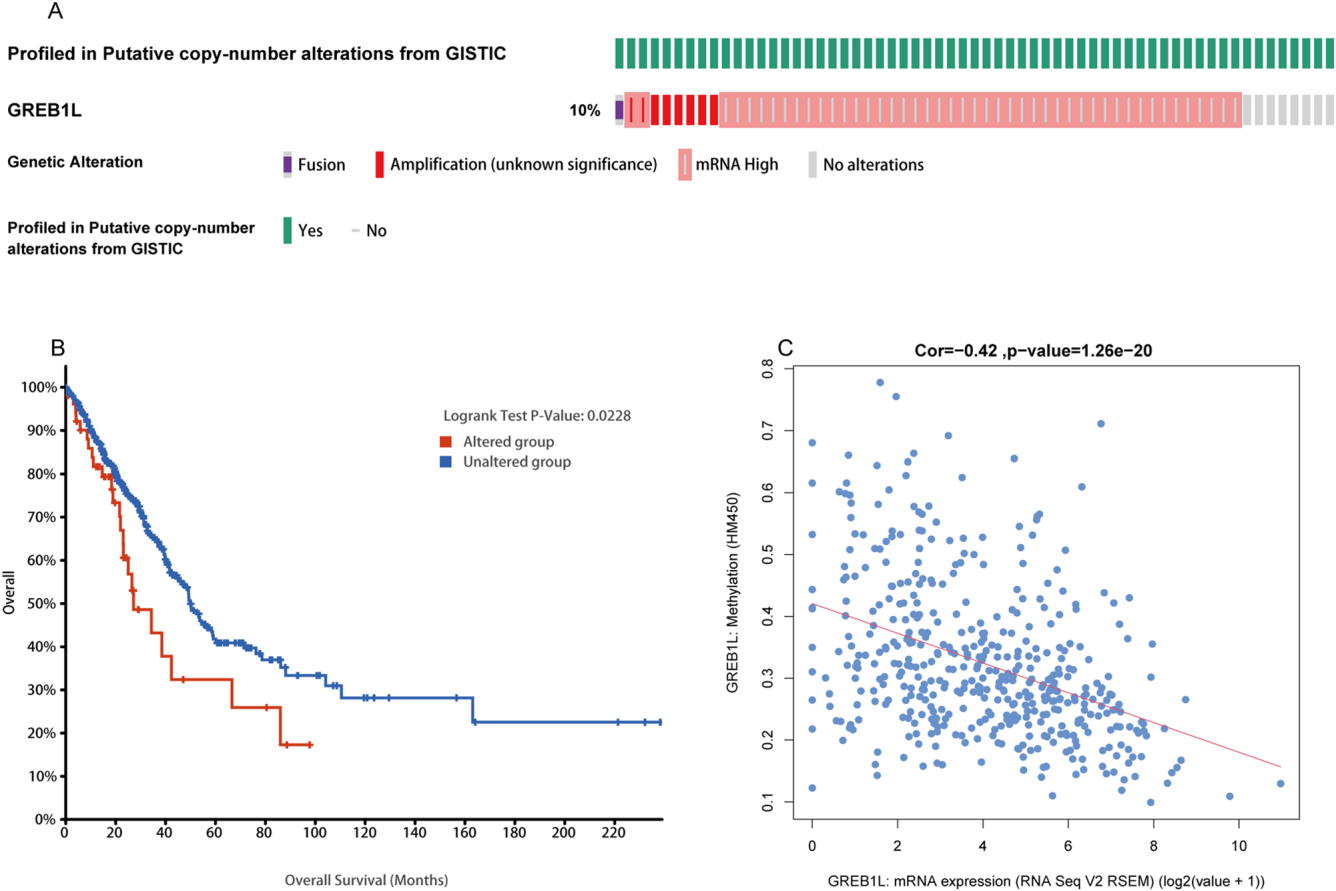
The gene alteration and methylation of GREB1L in LUAD. (**A**,**B**) Genetic alteration in GREB1L and its association with OS of LUAD patients; (**C**) The correlation between GREB1L methylation and its expression level.

**Figure 7:**
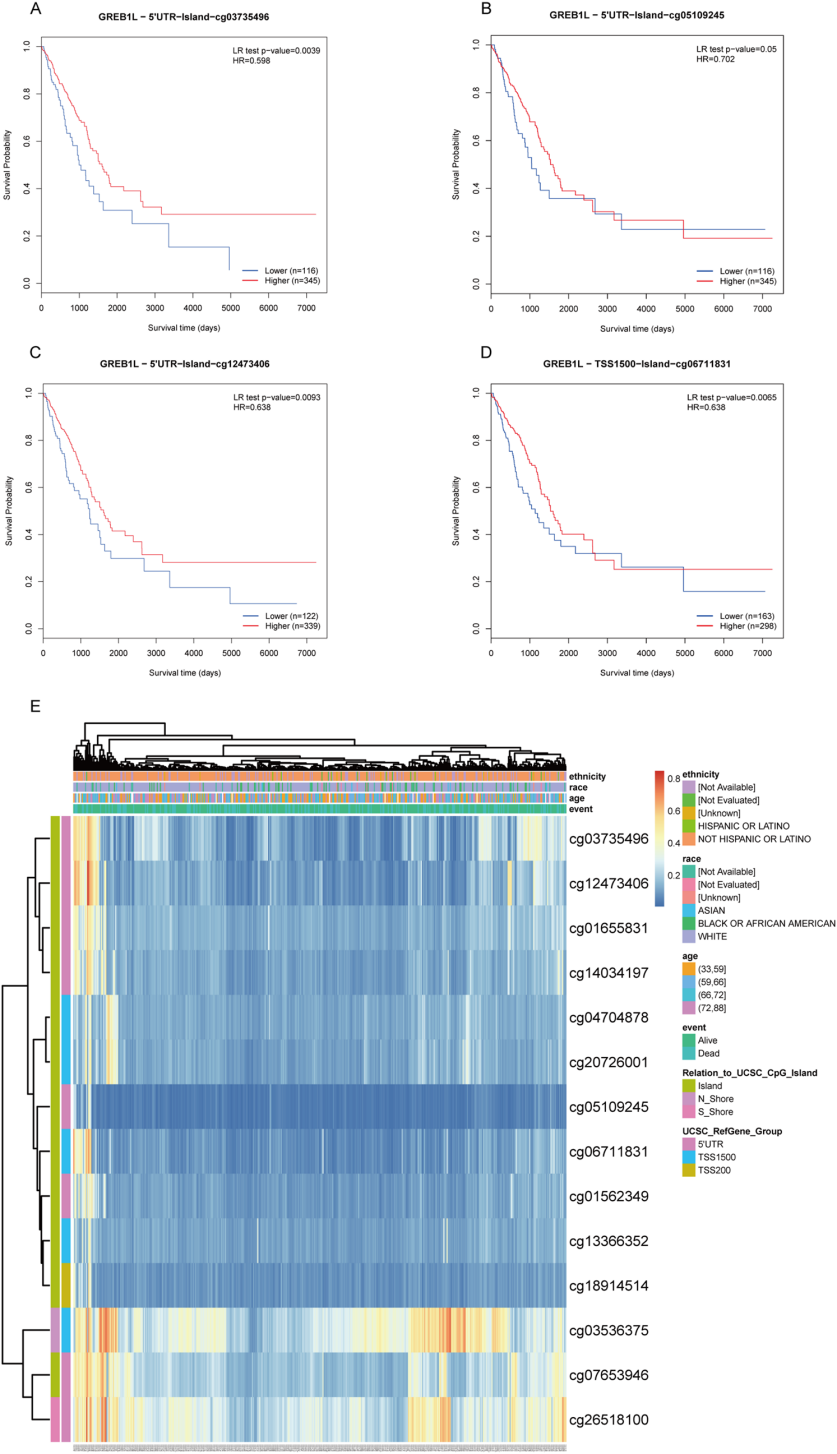
The MethSurv obtained the effect of hypomethylation level and GREB1L expression on prognosis in LUAD. (**A**–**D**) the Kaplan–Meier survival of the promoter methylation of GREB1L; (**E**) The visualization between the methylation level and the GREB1L expression. (software: MethSurv, version: MethSurv©2017, URL: https://biit.cs.ut.ee/methsurv/).

After validating the prognosis of GREB1L, we used cBioPortal to analyze the GREB1L expression and its mutation in LUAD. We analyzed genetic alteration in GREB1L and its associations with the prognosis of LUAD patients. As was shown in Fig. 6A, a high mutation rate of GREB1L was observed in LUAD patients. In the 501 sequenced LUAD patients, the genetic alteration was found in 52 LUAD patients and the mutation rate was 10%. Besides, the result showed that genetic alteration in GREB1L was associated with inferior OS of LUAD patients (Fig. 6B). These results implied that the genetic mutation of GREB1L could also affect LUAD patients’ prognosis. Considering that GSEA function enrichment analysis found that the GREB1L may participate in the methylation process, we then analyzed GREB1L methylation and GREB1L expression using the cBioPortal dataset. The results showed that GREB1L expression was highly negatively correlated with methylation (R =  − 0.42, p < 0.001) in LUAD (Fig. 6C). In addition, the MethSurv analysis showed that patients with low GREB1L methylation had a worse overall survival than patients with high GREB1L methylation (p < 0.05). We discovered that 4 CpG sites located on the CpG island indicated a poor prognosis, including cg03735496, cg05109245, cg12473406, and cg06711831 (Fig. 7A–D). Finally, the methylation level of GREB1L is low in LUAD by MethSurv (Fig. 7E).

### The correlation between GREB1L expression and the infiltration of the immune cells

**Figure 8:**
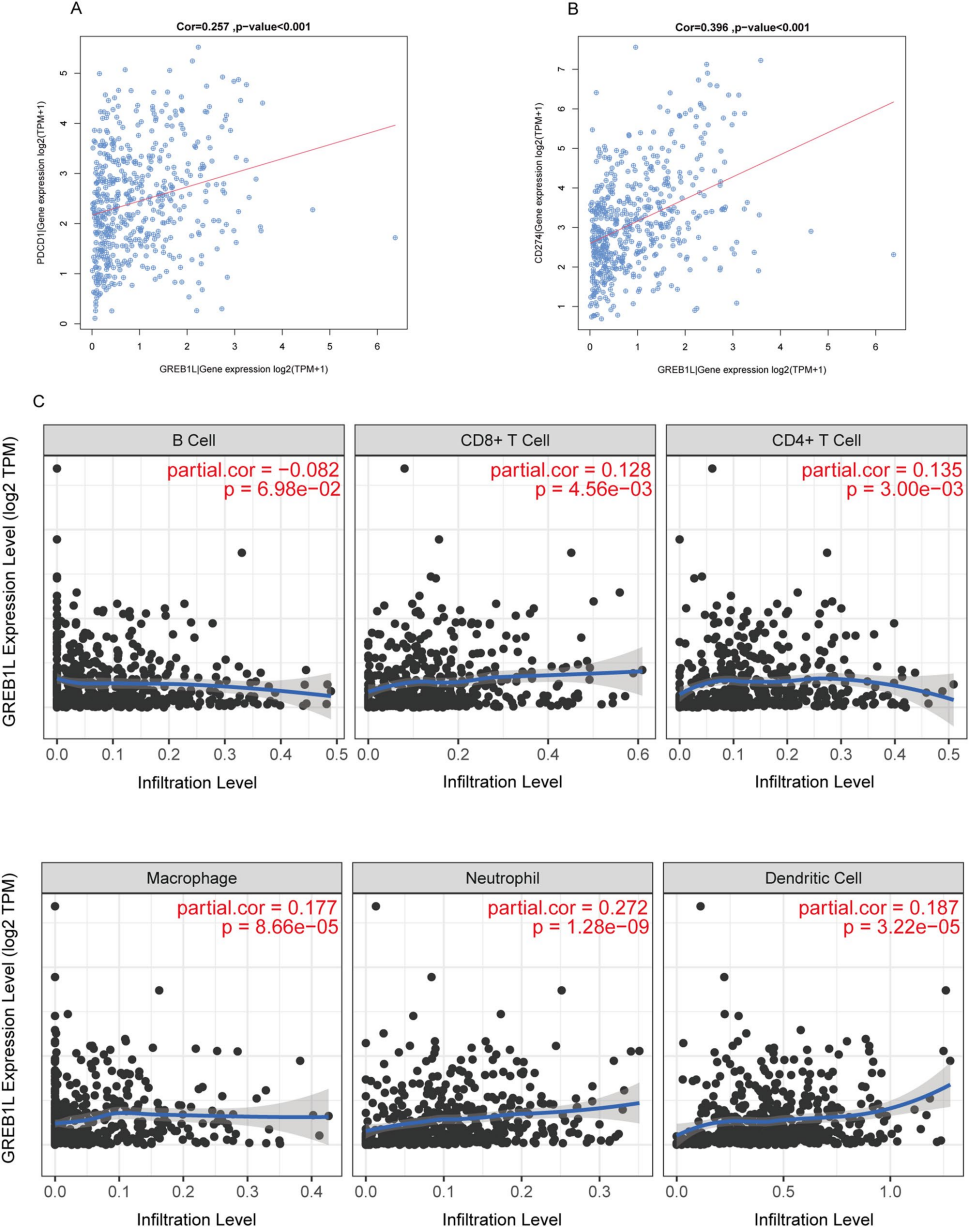
Correlation between PDCD1 (PD1), CD274 (PD-L1), immune cells infiltration, and GREB1L in LUAD. (**A**) The correlation of GREB1L expression and PDCD1 expression. (**B**) The correlation of GREB1L expression and CD274 expression; (**C**) GREB1L expression significantly positively correlated with the infiltration of CD8+ T cell, CD4+ T cell, macrophage, neutrophil, and dendritic cell, but not with the infiltration of B cell. (software: TIMER, version: TIMER1.0, URL: https://cistrome.shinyapps.io/timer/).

Considering that GSEA pathway enrichment analysis found that GREB1L may be associated with immune regulation, we further performed ssGSEA to examine the correlation between GREB1L expression and immune cell infiltration in LUAD. As was shown in Table 3, the activated CD4 T cell was positively correlated with GREB1L expression with Spearman correlation up to 0.349 (p < 0.001). We observed that GREB1L expression was also positively correlated with central memory CD4 T cell, effector memory CD4 T cell, central memory CD8 T cell, effector memory CD8 T cell, gamma delta T cell, immature B cell, memory B cell, regulatory T cell, T follicular helper cell, Type 1 T helper cell, Type 2 T helper cell, MDSC, Natural killer cell, Natural killer T cell, and neutrophil infiltration (all p < 0.05). Additionally, PD-1 (PDCD1) and PD-L1 (CD274) expression play an important role in tumor immune escape. They are also predictive markers for the therapeutic efficacy of immune checkpoint inhibitors (ICIs). The result showed that PD-1 and PD-L1 expression were positively correlated with GREB1L expression (R = 0.257 and 0.396, p < 0.001, respectively) in the LUAD-TCGA datasets (Fig. 8A,B). Finally, the analysis by TIMER software showed that the expression level of GREB1L was also positively correlated with the infiltration of CD8+ T cell (R = 0.128, p < 0.01), CD4+ T cell (R = 0.135, p < 0.01), macrophage (R = 0.177, p < 0.001), neutrophil (R = 0.272, p < 0.001), and dendritic cell (R = 0.187, p < 0.001), but not with the infiltration of B cell (R = 0.082, p = 0.0698) (Fig. 8C).

**Table 3:** The association between the expression level of GREB1L and the immune infiltration in the tumor microenvironment.

Immune cell	Spearman correlation	p-value
Activated CD4 T cell	0.349	< 0.001
Central memory CD4 T cell	0.103	0.018
Central memory CD8 T cell	0.207	< 0.001
Effector memory CD4 T cell	0.19	< 0.001
Effector memory CD8 T cell	0.232	< 0.001
Gamma delta T cell	0.209	< 0.001
Immature B cell	0.121	0.005
Memory B cell	0.245	< 0.001
Regulatory T cell	0.222	< 0.001
T follicular helper cell	0.089	0.042
Type 1 T helper cell	0.159	< 0.001
Type 17 T helper cell	− 0.112	0.010
Type 2 T helper cell	0.162	< 0.001
MDSC	0.124	0.004
Monocyte	− 0.099	0.024
Natural killer cell	0.156	< 0.001
Natural killer T cell	0.245	< 0.001
Neutrophil	0.094	0.031

## Discussion

Lung adenocarcinoma is a malignant tumor characterized by uncontrolled growth of cells in the lung and bronchus^2^. The clinical LUAD outcomes are far from satisfactory using current treatments. Therefore, it is crucial to find stable potential biomarkers to predict prognosis and guide individualized therapies. By analyzing TCGA datasets, we found GREB1L was overexpressed in LUAD and correlated with poor OS. Our result showed that it is highly expressed in LUAD by the GSE140343 and the HPA. Meanwhile, the prognostic value of GREB1L was validated using three GEO datasets (GSE13213, GSE30219, and GSE50081). Furthermore, we confirmed that GREB1L was an independent prognostic factor for LUAD using TCGA and GSE13213 datasets. Functional enrichment analyses found that GREB1L was associated with cell cycle, immune regulation, and methylation. We also indicated that GREB1L genetic alteration and hypomethylation were associated with inferior OS in LUAD. Further analyses showed that GREB1L overexpression was positively associated with immune cell infiltration. GREB1L expression positively correlated with the expression of PD-1 and PD-L1. Thus, our study provides new insights into understanding the potential roles of GREB1L in tumor microenvironment and its potential use as cancer therapeutic and prognostic biomarker.

GREB1L is an important paralog of GREB1 (Growth Regulating Estrogen Receptor Binding 1). At present, the biological mechanism of GREB1L in tumors is only little understood. Our GSEA function enrichment analysis found that GREB1L may participate in cell cycle, immune regulation, and methylation in LUAD. Other function enrichment results are related to the DNA conformation change, protein-DNA complex subunit organization, double-strand break repair, protein-DNA complex assembly, DNA packaging, nuclear chromatin, and chromosomal region, which need to be confirmed by further study. Interestingly, GSEA pathwayanalysis found that GREB1L is related to the cell cycle, microRNAs in cancer, JAK-STAT signaling pathway, cytokine-cytokine receptor interaction, Wnt signaling pathway, Ras signaling pathway, and cGMP-PKG signaling pathway. These results suggested that GREB1L may play an important role in the tumorigenesis, immune regulation, and methylation process. Therefore, targeting GREB1L seems to be an alternative strategy for tumor therapy.

DNA methylation is a common epigenetic mechanism present in all forms of cancer^12^. Promoter methylation accompanies gene silencing^13^. In this study, the GSEA-function analysis showed that GREB1L participates in the epigenetic regulation of gene expression and gene silencing. A previous study also confirmed that the methylation level of GREB1L is related to immune response and cytolysis in gastric adenocarcinoma, suggesting that it may be a new prediction and prognostic biomarker that aids in the therapy and predicts the overall survival possibility in patients with gastric adenocarcinoma^11^. In the study, we further explored the mechanism of GREB1L overexpression in LUAD, and our results showed that GREB1L overexpression might be related to GREB1L hypomethylation (R =  − 0.42, p < 0.001). Interestingly, GREB1L methylation was associated with the prognosis of LUAD, and hypomethylated patients have worse overall survival, which is consistent with the prognostic value of the mRNA expression of this gene. Although many mechanisms can give rise to elevated gene expression, hypomethylation is one of the main regulatory mechanisms of gene expression.

In recent years, immunotherapy to boost T cell functionality in tumors is rapidly becoming standard treatment. The immunotherapy focus has been on recruiting tumor-infiltrating T cells^14,15^. CD4+ T cells secrete a variety of cytokines that have direct effector functions and activate other immune cells (such as B cells and CD8 T Cells)^16,17^. In lung cancer, tumor-infiltrating CD4+ T cells play an essential role in the immune response^18^. CD4+ T cells affect tumors by allowing CD8 + T cells entry to tumor sites or mucosa^19,20^. Furthermore, they are also required for the inhibition of angiogenesis at tumor sites^21^. Our GSEA pathway analysis result indicated that GREB1L expression was significantly involved in “JAK-STAT Signaling pathway” and “cytokine-cytokine receptor interaction”. According to previous study, Janus kinase-signal transducer and activator of transcription (JAK-STAT) signaling mediates almost all immune regulatory processes, including those that are involved in tumor cell recognition and tumor-driven immune escape^22^. Additionally, cytokines are major regulators of the innate and adaptive immune systems that allow cells of the immune systems to communicate over short distances in paracrine and autocrine fashion^23^. Our results also showed that the GREB1L expression was positively correlated with the immune infiltration level of T cells, neutrophils, NK cells, MDSC, and dendritic cells, especially in activated CD4 T cell, central memory CD4 T cell, effector memory CD4 T cell, central memory CD8 T cell, and effector memory CD8 T cell. Similarly, TIMER analysis showed that GREB1L expression was positively correlated with the infiltration of CD4+ T cell, CD8+ T cell, macrophage, neutrophil, and dendritic cell. These results suggested that the expression level of GREB1L may indicate the level of immune infiltration level of tumor cells, providing a reference for the immunotherapy of LUAD. Interestingly, our results showed that GREB1L expression was also positively correlated with PD-1 and PD-L1 expression in LUAD. High expression of PD-L1 has been detected in many tumors, including NSCLC, and is associated with poor prognosis^24^. Previous studies indicated that tumor patients with overexpression of PD-L1 have better clinical outcomes in anti-PD-L1 therapy, while some patients with low expression of PD-L1 have an inferior effect^25^. Therefore, our result demonstrated that GREB1L might affect immune cell infiltration and immunotherapy efficacy, which makes them a predictive biomarker for immunotherapy in LUAD patients.

Although this study improved our understanding of GREB1L in LUAD, there were some limitations. Firstly, the correlation between GREB1L mRNA and protein expression should be verified using cellular and clinical experiments. Secondly, we also cannot clearly estimate the direct mechanisms of GREB1L involved in the development of LUAD. Thirdly, the specific role of GREB1L in the development of LUAD should be comprehensively elucidated. Further experimental researches are needed to confirm it. In order to further investigate the mechanism of GREB1L in LUAD, we plan to conduct cellular experiments in the near future.

## Conclusion

In conclusion, our findings suggested that GREB1L overexpression is an independent adverse prognostic factor in LUAD. GREB1L DNA amplification and promoter demethylation might contribute to GREB1L upregulation, and GREB1L DNA amplification, genetic alteration, and hypomethylation are associated with poor outcome. Besides, GREB1L mediates immune cell infiltration in the tumor microenvironment. This study demonstrated GREB1L as a prognostic biomarker for LUAD, highlighting its potential as a predictive biomarker and an immunotherapy target.

## Materials and methods

### Data acquisition

Datasets from the TCGA database were included: gene expression data (HTSeq-Counts and HTSeq-FPKM) and the corresponding detailed clinical data from LUAD samples. We downloaded these data from the UCSC Xena browser (version 07-20-2019, https://xenabrowser.net/datapages/). Cases with insufficient or missing data were removed from subsequent data processing. Finally, there are 497 LUAD cases and 57 normal cases in our study. LUAD patients were classified into low- and high- GREB1L expression groups according to the median GREB1L expression value. GREB1L expression data and clinical information from datasets GSE13213, GSE30219, and GSE50081 were downloaded from the GEO database and validated for survival analyses. Our datasets used in the study were consistent with the publication guidelines provided by the online database. The ethics approval and informed consent were not required.

### Over-expression of GREB1L in LUAD patients

We explored the expression differences of GREB1L in LUAD patients between non-pair and pair tissues. The GSE140343 and the Human Protein Atlas (https://www.proteinatlas.org) analyzed mRNA expression and protein expression to examine the expression of GREB1L in LUAD. Antibody HPA 044218 was used for immunohistochemistry staining of GREB1L. Besides, the ROC curve was used to evaluate the diagnostic value of GREB1L using the pROC R package (version:1.16.2, https://cran.r-project.org/web/packages/pROC/index.html) and ggplot2 R package (version:3.3.2, https://cran.r-project.org/web/packages/ggplot2/index.html).

### Role of GREB1L in LUAD patient survival

The survival curve was generated by the survminer R package (version 0.4.8, https://cran.r-project.org/web/packages/survminer/index.html) and the survival R package (version 0.1.3, https://cran.r-project.org/web/packages/survivalAnalysis/index.html).

### Construction and evaluation of the nomogram

The rms R package (Version:6.0-1, https://cran.r-project.org/web/packages/rms/index.html) was performed to generate a nomogram. C-index and calibration curve were performed by the Hmisc R package (Version:4.4-1, https://cran.r-project.org/web/packages/Hmisc/index.html). In our study, C-index was performed to determine the discrimination of nomogram and used a bootstrap method with 1000 resamples to calculated C-index.

### Function and pathway analysis by gene set enrichment analysis (GSEA)

Expression datasets (HTSeq-Counts) were compared between low- and high- GREB1L expression groups to identify the differentially expressed genes (DEGs) using the DESeq2 R package (version 1.28.1, http://www.bioconductor.org/packages/release/bioc/html/DESeq2.html). Gene set enrichment analysis (GSEA) is a calculation method that determines whether a set of prior defined genes show statistically significant and consistent differences between two biological states^26^. In this study, GSEA was performed using the clusterProfiler R package (version 3.18.0, http://bioconductor.org/packages/release/bioc/html/clusterProfiler.html) to demonstrate the significant functions and pathways between the low- and high-GREB1L groups. The expression level of GREB1L was used as a phenotype label. An adjusted p-value < 0.05, normalized enrichment score (|NES|) > 1, and false discovery rate (FDR) < 0.25 were considered as significant difference.

### Analysis of GREB1L mutation, methylation, and prognosis

The mutation data of GREB1L was obtained from the cBioPortal (https://www.cbioportal.org/) web platform. In the study, we explored the genomic profiles of GREB1L with a z-score threshold ± 1.5. Genetic mutations in GREB1L and their association with overall survival was carried out to identify its prognostic value. GREB1L methylation data were also downloaded from the cBioPortal (https://www.cbioportal.org/) web platform. The correlation between the GREB1L gene expression and the GREB1L methylation level (Spearman correlation) was conducted. Moreover, we analyzed the prognostic value of the GREB1L methylation level in LUAD by MethSurv online tool (version MethSurv©2017, https://biit.cs.ut.ee/methsurv/)^27^. It is a web tool to provide survival analysis based on DNA methylation biomarkers using TCGA data.

### Analysis of immune infiltration and its correlation with GREB1L expression

To elucidate the correlation between GREB1L and the immune cells infiltration level, ssGSEA (single-sample Gene Set Enrichment Analysis) from the GSVA package (version 1.36.3, http://www.bioconductor.org/packages/release/bioc/html/GSVA.html) was used to examine the relative proportions of different kinds of immune cells infiltration level in tumor microenvironments to study the relationship of GREB1L and immune infiltration. The immune reference set was downloaded from the web platform (10.1016/j.celrep.2016.12.019)^28^. The relative levels of 28 types of tumor-infiltrating immune cells in the immunocyte signatures, including 782 genes for predicting in individual tissue samples. Based on the 28 types of immunocytes’ signature genes in the literature, every immunocyte’s relative enrichment score was quantified from each tumor sample’s gene expression profile^29^. The correlation between GREB1L and the immune cells infiltration level was performed by Spearman correlation. Finally, TIMER software was performed to validate the correlation between the different GREB1L expression levels and the infiltration of the immune cells in LUAD samples from the TCGA databas^30^.

### Statistical analysis

The statistical analysis was analyzed using the SPSS (version 26.0) and R (version 4.0.2, https://www.R-project.org/). The Wilcoxon signed-rank test and Wilcoxon rank-sum test were performed to investigate the expression of GREB1L in paired and non-paired samples, respectively. The Wilcoxon signed-rank test was used to analyze the relations between GREB1L expression and clinical features. The univariate and multivariate analyses using the Cox regression model were carried out to evaluate death risk, including gender, age, number pack years smoked, T stage, N stage, M stage, pathological stage, and GREB1L expression. All the tests were two-sided, and p-values < 0.05 were considered statistically significant.

## Supplementary Information

Supplementary Information.

## Data Availability

The datasets used and/or analyzed during the current study are available from the corresponding author on reasonable request.

## References

[1] Global Burden of Disease Cancer Consortium (2019). Global, regional, and national cancer incidence, mortality, years of life lost, years lived with disability, and disability-adjusted life-years for 29 cancer groups, 1990 to 2017: A systematic analysis for the global burden of disease study. JAMA Oncol..

[2] Siegel RL, Miller KD, Jemal A (2020). Cancer statistics, 2020. CA Cancer J. Clin..

[3] Zheng M (2016). Classification and pathology of lung cancer. Surg. Oncol. Clin. N. Am..

[4] Kris MG (2014). Using multiplexed assays of oncogenic drivers in lung cancers to select targeted drugs. JAMA.

[5] Aris VM (2004). Noise filtering and nonparametric analysis of microarray data underscores discriminating markers of oral, prostate, lung, ovarian and breast cancer. BMC Bioinform..

[6] Fridman WH, Pages F, Sautes-Fridman C, Galon J (2012). The immune contexture in human tumours: Impact on clinical outcome. Nat. Rev. Cancer.

[7] Becht E, Giraldo NA, Dieu-Nosjean M-C, Sautès-Fridman C, Fridman WH (2016). Cancer immune contexture and immunotherapy. Curr. Opin. Immunol..

[8] Boissel S (2018). Genomic study of severe fetal anomalies and discovery of GREB1L mutations in renal agenesis. Genet. Med..

[9] De Tomasi L (2017). Mutations in GREB1L cause bilateral kidney agenesis in humans and mice. Am. J. Hum. Genet..

[10] Schrauwen I (2018). De novo variants in GREB1L are associated with non-syndromic inner ear malformations and deafness. Hum. Genet..

[11] Hu S, Yin X, Zhang G, Meng F (2019). Identification of DNA methylation signature to predict prognosis in gastric adenocarcinoma. J. Cell Biochem..

[12] Klutstein M, Nejman D, Greenfield R, Cedar H (2016). DNA methylation in cancer and aging. Cancer Res..

[13] Yamashita K, Hosoda K, Nishizawa N, Katoh H, Watanabe M (2018). Epigenetic biomarkers of promoter DNA methylation in the new era of cancer treatment. Cancer Sci..

[14] Oja AE (2018). Functional heterogeneity of CD4+ tumor-infiltrating lymphocytes with a resident memory phenotype in NSCLC. Front. Immunol..

[15] Bruno TC (2017). Antigen-presenting intratumoral B cells affect CD4 (+) TIL phenotypes in non-small cell lung cancer patients. Cancer Immunol. Res..

[16] Kamphorst AO, Ahmed R (2013). CD4 T-cell immunotherapy for chronic viral infections and cancer. Immunotherapy.

[17] Swain SL, McKinstry KK, Strutt TM (2012). Expanding roles for CD4 (+) T cells in immunity to viruses. Nat. Rev. Immunol..

[18] Hiraoka K (2006). Concurrent infiltration by CD8+ T cells and CD4+ T cells is a favourable prognostic factor in non-small-cell lung carcinoma. Br. J. Cancer.

[19] Bos R, Sherman LA (2010). CD4+ T-cell help in the tumor milieu is required for recruitment and cytolytic function of CD8+ T lymphocytes. Cancer Res..

[20] Nakanishi Y, Lu B, Gerard C, Iwasaki A (2009). CD8 (+) T lymphocyte mobilization to virus-infected tissue requires CD4 (+) T-cell help. Nature.

[21] Rakhra K (2010). CD4 (+) T cells contribute to the remodeling of the microenvironment required for sustained tumor regression upon oncogene inactivation. Cancer Cell.

[22] Owen KL, Brockwell NK, Parker BS (2019). JAK-STAT signaling: A double-edged sword of immune regulation and cancer progression. Cancers.

[23] Berraondo P (2018). Cytokines in clinical cancer immunotherapy. Br. J. Cancer.

[24] Ohaegbulam KC, Assal A, Lazar-Molnar E, Yao Y, Zang X (2015). Human cancer immunotherapy with antibodies to the PD-1 and PD-L1 pathway. Trends Mol. Med..

[25] Patel SP, Kurzrock R (2015). PD-L1 expression as a predictive biomarker in cancer immunotherapy. Mol. Cancer Ther..

[26] Subramanian A (2005). Gene set enrichment analysis: A knowledge-based approach for interpreting genome-wide expression profiles. Proc. Natl. Acad. Sci. U.S.A..

[27] Modhukur V (2018). MethSurv: A web tool to perform multivariable survival analysis using DNA methylation data. Epigenomics.

[28] Charoentong P (2017). Pan-cancer immunogenomic analyses reveal genotype-immunophenotype relationships and predictors of response to checkpoint blockade. Cell Rep..

[29] Bindea G (2013). Spatiotemporal dynamics of intratumoral immune cells reveal the immune landscape in human cancer. Immunity.

[30] Li T (2017). TIMER: A web server for comprehensive analysis of tumor-infiltrating immune cells. Cancer Res..

