# Supplementary figures and images for: GREB1L overexpression correlates with prognosis and immune cell infiltration in lung adenocarcinoma
Source: Sci Rep. 2021 Jun 24;11:13281. doi: 10.1038/s41598-021-92695-x (PMC8225624; doi:10.1038/s41598-021-92695-x)

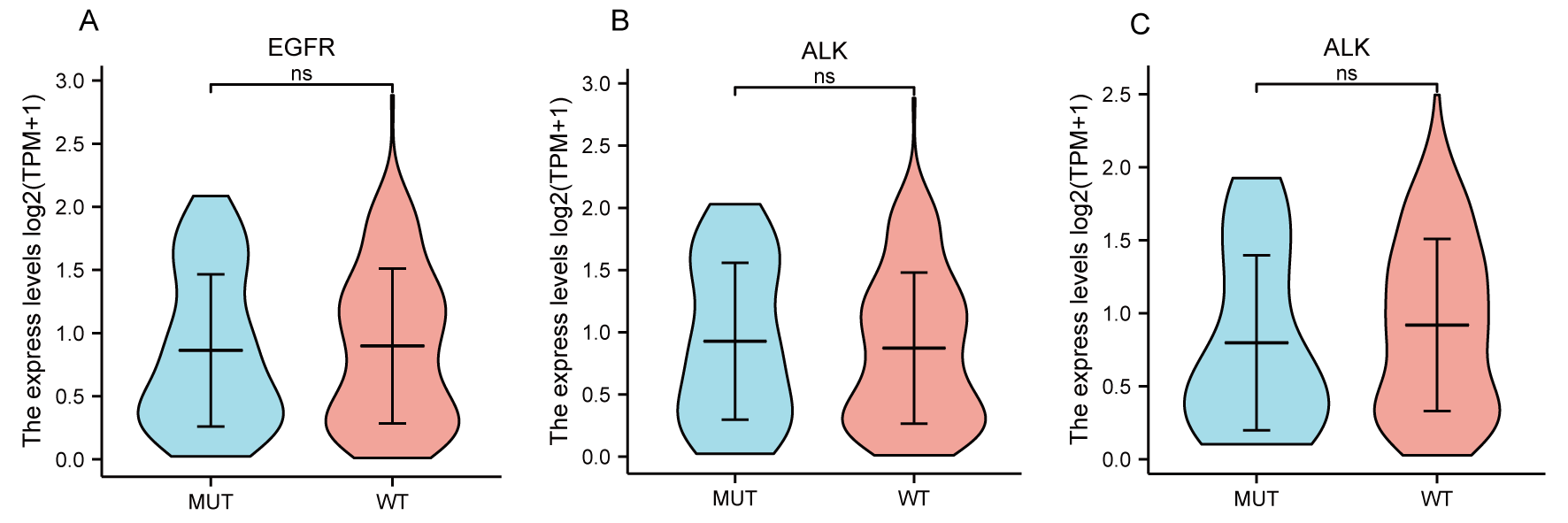

Supplement: Supplementary file 1 — Supplementary Information. [file 41598_2021_92695_MOESM1_ESM.tif]
